# pH-Sensitive and Charge-Reversal Polymeric Nanoplatform Enhanced Photothermal/Photodynamic Synergistic Therapy for Breast Cancer

**DOI:** 10.3389/fbioe.2022.836468

**Published:** 2022-02-18

**Authors:** Wenyan Wang, Zimu Li, Xiaozhong Nie, Wenfeng Zeng, Yi Zhang, Yimin Deng, Hongzhong Chen, Xiaowei Zeng, Hualin Ma, Yi Zheng, Nansha Gao

**Affiliations:** ^1^ Institute of Pharmaceutics, School of Pharmaceutical Sciences (Shenzhen), Sun Yat-sen University, Shenzhen, China; ^2^ School of Food and Drug, Shenzhen Polytechnic, Shenzhen, China; ^3^ Shenzhen Key Laboratory of Kindey Diseases, Department of Nephrology, Shenzhen People’s Hospital (The Second Clinical Medical College, Jinan University, The First Affiliated Hospital, Southern University of Science and Technology), Shenzhen, China; ^4^ Central Laboratory, University of Chinese Academy of Sciences-Shenzhen Hospital, Shenzhen, China

**Keywords:** cancer nanotechnology, charge-reversal, photodynamic therapy, photothermal therapy, pH-sensitive

## Abstract

As reported, breast cancer is one of the most common malignancies in women and has overtaken lung cancer as the most commonly diagnosed cancer worldwide by 2020. Currently, phototherapy is a promising anti-tumor therapy due to its fewer side effects, less invasiveness, and lower cost. However, its application in cancer therapeutics is limited by the incomplete therapeutic effect caused by low drug penetration and monotherapy. Herein, we built a charge-reversal nanoplatform (Ce6-PLGA@PDA-PAH-DMMA NPs), including polydopamine (PDA) and chlorin e6 (Ce6) for enhancing photothermal/photodynamic synergistic therapy. The PAH-DMMA charge-reversal layer enabled Ce6-PLGA@PDA-PAH-DMMA NPs to have long blood circulation at the normal physiological environment and to successfully realize charge reversal under the weakly acidic tumor microenvironment, improving cellular uptake. Besides, *in vitro* tests demonstrated that Ce6-PLGA@PDA-PAH-DMMA NPs had high photothermal conversion and greater anti-tumor activity than no charge-reversal nanoparticles, which overcame the limited tumor therapeutic efficacy of PTT or photodynamic therapy alone. Overall, the design of pH-responsive and charge-reversal nanoparticles (Ce6-PLGA@PDA-PAH-DMMA NPs) provided a promising approach for synergistic PTT/PDT therapy against breast cancer.

## Introduction

According to World Health Organization (WHO) statistics, almost 19.3 million cancer patients have been diagnosed, and close to 10.0 million deaths have occurred in 2020 worldwide (Ferlay et al., 2021; Sung et al., 2021). Every fifth people in the world have cancer in their lifetime, reflecting that cancer is one of the most critical public health issues. Based on the population growth and aging, the global burden of cancer incidence and mortality increases rapidly (Gersten and Wilmoth, 2002; Omran, 2005). Amazingly, breast cancer in women has become the most frequently diagnosed cancer, exceeding lung cancer (Printz, 2021).

Nowadays, conventional clinical cancer therapies for breast cancer, such as surgery, chemotherapy, and radiotherapy, have high toxicity and aggressiveness, low selectivity, and low efficacy (Dolmans et al., 2003; Bonavida and Chouaib, 2017; Cai et al., 2019). In recent years, some new approaches, including gene therapy, immunotherapy, and phototherapy, have been developed to improve breast cancer treatment (Wang et al., 2021). Phototherapy consisting of photodynamic therapy (PDT) and photothermal therapy (PTT) is a promising option for anti-breast cancer therapy because of its apparent advantages, such as fewer side effects, less invasiveness, high curative rate, and lower cost (Zeng et al., 2020; Zou et al., 2021). In PDT, the photosensitizer could transfer energy to oxygen to generate reactive oxygen species (ROS) after irradiating specific light wavelengths, killing neighboring cancer cells (Dolmans et al., 2003; Lucky et al., 2015). PTT can efficiently damage the tumor tissues by transforming near-infrared NIR light energy into heat (Zhang et al., 2020). However, monotherapy of PTT or PDT is limited by incomplete anti-tumor treatment caused by insufficient light penetration, heat resistance, or hypoxia (Jaque et al., 2014; Wang W. et al., 2020). Moreover, low drug penetration of the tumor tissue and low drug uptake of cancer cells weaken the therapeutic efficacy of monotherapy (Yang et al., 2020; Cao et al., 2021). Studies have found that the combination of PDT and PTT had a synergistic effect (Sun et al., 2021; Zhang et al., 2021). Under heat generated by the low-energy laser, the photothermal agent could not only improve the permeability of the cell membrane and increase the cellular uptake of the photosensitizer (Shi et al., 2013), but also increase the blood flow of the tumor tissue and relieve the hypoxia (Song et al., 2015).

It has been reported that nanoplatform-based phototherapy received considerable attention for breast cancer treatment (Dang and Guan, 2020; Shi et al., 2020; Huang et al., 2021; Yang et al., 2021). Nanoparticles (NPs) loading the photosensitizer and the photothermal agent could efficiently deliver to the tumor tissue *via* enhanced permeability and retention (EPR) effect (Zeng et al., 2020). Besides, NPs with positive surface charges were easier to be taken up by cancer cells with negative charge due to the charge interaction, while negatively charged NPs could effectively reduce the immune clearance, increasing circulation time (Liu et al., 2019). Thus, NPs with the charge-reversal layer could promote cellular uptake and reduce the side effects (Gao et al., 2019). In this study, we engineered the pH-responsive and charge-reversed NPs (Ce6-PLGA@PDA-PAH-DMMA NPs) to achieve synergistic PTT/PDT therapy against breast cancer.

**FIGURE 1:**
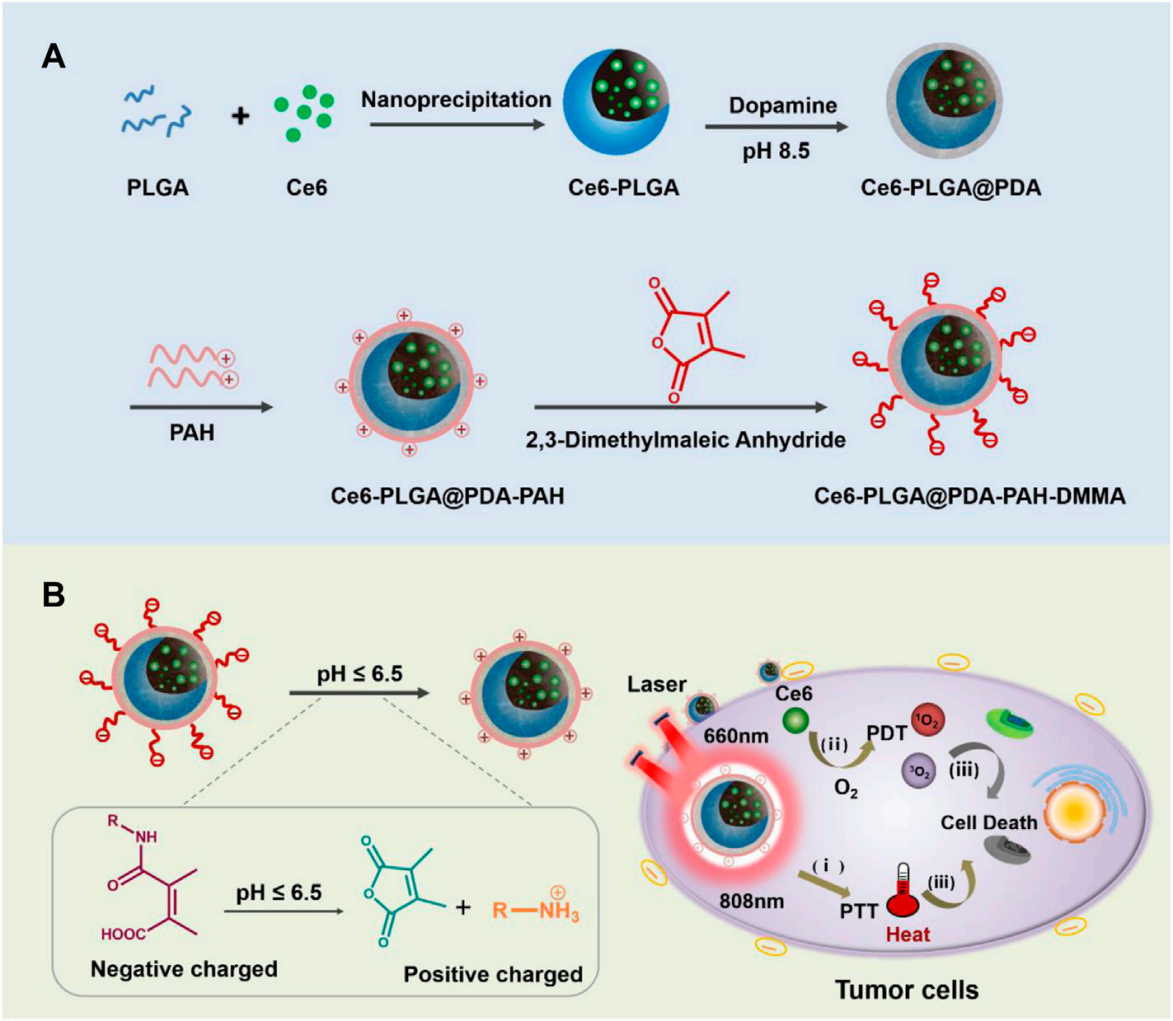
**(A)** Schematic diagram of the preparation progress of Ce6-PLGA@PDA-PAH-DMMA NPs. **(B)** Ce6-PLGA@PDA-PAH-DMMA NPs applied to PTT/PDT synergistic breast cancer therapy.

As we all know, poly-lactic-co-glycolic acid (PLGA) has been widely applied in biomedical applications based on its excellent biocompatibility and biodegradability (Thamake et al., 2012; Peng et al., 2018). Meanwhile, PLGA has a good loading content and can satisfy the requirement of drug delivery (Park et al., 2012; Zeng et al., 2013). In this design, chlorin e6 (Ce6) was loaded into PLGA. Polydopamine (PDA), an excellent photothermal agent with good biocompatibility (Zeng et al., 2018; Li et al., 2021a; Li et al., 2021b; Hou et al., 2021), was applied to decorate Ce6-PLGA NPs. Then, positively charged poly-(allyamine) (PAH) produced by amino groups was connected to Ce6-PLGA@PDA NPs. Finally, the negatively charged polymer dimethyl-maleic acid (DMMA) was attached to shield the positive charge of PAH, assembling the PAH-DMMA charge-reversal layer (Feng et al., 2016; Xu et al., 2017). The amide bond of DMMA would break and expose positively charged amino groups under the weakly acidic tumor microenvironment (Li et al., 2021c), resulting in the surface charge reversal of Ce6-PLGA@PDA-PAH-DMMA NPs from negative to positive, facilitating cellular endocytosis and accumulating at the tumor tissue. Subsequently, after irradiating with the 808-nm and 660-nm lasers, the NPs produced a large amount of heat and toxic ROS, damaging the tumor tissue (Cui et al., 2019; Wang Y. et al., 2020) (Figure 1) In conclusion, the NPs (Ce6-PLGA@PDA-PAH-DMMA NPs) we developed offer a promising approach for efficient cancer synergistic PTT/PDT and have extensive clinical application prospects.

## Methods

### Synthesis of Ce6-PLGA@PDA NPs

First, Ce6 (5 mg) and PLGA copolymers (200 mg) were dissolved in acetone (16 ml) and added into TPGS aqueous solution (200 ml, 0.03%). The solution was stirred overnight in the dark. The final reactant was centrifuged (20,000 rpm, 20 min) to obtain Ce6-PLGA NPs. Afterward, dopamine (15 mg) was dispersed in 150 ml of Tris-HCl buffer and stirred in darkness (12 h). Ce6-PLGA@PDA NPs were gained by centrifugation. Synthesis of Ce6-PLGA@PDA-PAH-DMMA NPs.

Briefly, Ce6-PLGA@PDA NPs and PAH were added in Tris-HCl buffer and stirred overnight. The PLGA@PDA-PAH NPs were obtained by centrifuging (20,000 rpm, 20 min) and washing twice. Subsequently, Ce6-PLGA@PDA-PAH NPs were dispersed in DMSO (5 ml) and then DMMA (30 mg) and triethylamine (130 μl) were added to stir for 24 h. Finally, Ce6-PLGA@PDA-PAH-DMMA NPs were obtained by centrifugation. The Ce6-PLGA@PDA-PEG NPs were prepared by replacing PAH with PEG-NH_2_.

### Characterization of NPs

The morphology was determined by a transmission electron microscope (TEM, JEOL Ltd., Japan). Fourier transform infrared (FTIR) absorption spectrometry was used to observe the chemical bond connection after modification. X-ray photoelectron spectroscopy (XPS; ESCALAB 250Xi, Japan) was applied to analyze the surface chemistry. A UV-vis spectroscopy (LAMBDA365) was employed to evaluate the loading of Ce6. The 808-nm laser (BOHR-the 808-FCIR8) was used for photothermal therapy. An IR thermal imaging camera was used to record the temperature changes. The production of ROS by the cells and the NPs cellular uptake efficiency were detected by confocal laser scanning microscopy (CLSM). The cell viability was analyzed by a Spectra M2 plate reader (SpectraMax®i3x). An IR thermal camera (Fluke Ti480) was used to examine *in vitro* photothermal performance.

### *In Vitro* Photothermal Property

For analyzing the photothermal property of different NPs, various solutions (PBS, Ce6-PLGA NPs, Ce6-PLGA@PDA NPs, Ce6-PLGA@PDA-PAH NPs, and Ce6-PLGA@PDA-PAH-DMMA NPs) were irradiated for 10 min *via* the 808-nm laser. To test the concentration effect of the NPs, different concentrations of Ce6-PLGA@PDA-PAH-DMMA NPs (0.25, 2.5, 12.5, 25, and 50 μg/ml) were irradiated for 10 min (808 nm, 1.5 W/cm^2^). The Ce6-PLGA@PDA-PAH-DMMA NPs solution (25 μg/ml) was irradiated for 10 min under various power densities. Finally, to observe the photothermal stability of the Ce6-PLGA@PDA-PAH-DMMA NPs solution (25 μg/ml), the solution was irradiated for 10 min in five times laser on/off cycles (1.5 W/cm^2^).

### Cellular Uptake Experiments

MCF-7 cells were cultured in confocal dishes for 12 h in an incubator (37°C, 5% CO_2_). Then, medium, including various Ce6 preparations labeled with Cy5 (Ce6 = 2 μg/ml), was added. After co-incubation for 2 h, the cells were fixed with 4% paraformaldehyde and dyed with DAPI. The results were analyzed by CLSM (DAPI and Cy5 fluorescent channels). Moreover, MCF-7 cells were seeded in plates in an incubator (37°C, 5% CO_2_) for 24 h. Then, the medium including Ce6-PLGA@PDA-PAH-DMMA-Cy5 NPs and Ce6@PDA-PEG-Cy5 NPs was added, followed by incubating for 2 h. Finally, the MCF-7 cells were collected for flow cytometer analysis.

### *In Vitro* Cytotoxicity Assay

#### Dark Toxicity

First, 4T1 or MCF-7 cells (5×10^3^ per well) were cultured for 12 h in an incubator (37°C, 5% CO_2_) until complete adhesion. Then, the medium containing different concentrations of Ce6-PLGA@PDA-PAH-DMMA NPs (7.8, 15.6, 31.3, 63.5, 125, and 250 μg/ml) at pH 7.4 was added.

#### The Photo-Cytotoxicity

The cells (5 × 10^3^ per well) were cultured for 12 h in an incubator (37°C, 5% CO_2_) until complete adhesion. Then, some medium, containing free Ce6, Ce6-PLGA@PDA-PEG NPs, Ce6-PLGA@PDA-PEG NPs, and Ce6-PLGA@PDA-PAH-DMMA NPs, was added separately, each group with concentrations of Ce6 (1, 2, and 4 μg/ml). After incubation for 6 h, the cells were treated with the 660-nm laser (200 mV/cm^2^, 5 min) and cultured for another 24 h.

#### Combination Therapy

For the combined therapy experiment, cells (5 × 10^3^ per well) were cultured for 12 h. Then, some medium containing PBS, Ce6-PLGA@PDA-PEG NPs, and Ce6-PLGA@PDA-PAH-DMMA NPs (Ce6 = 2 μg/ml) was added, with or without irradiation by laser. After being co-cultured for 6 h, the drug-containing medium was removed. Then, the related groups were treated with the 660-nm laser (200 mV/cm^2^, 5 min) or the 808-nm laser (1.5 W/cm^2^, 10 min), or both, and cultured for another 24 h.

#### *In Vitro* ^1^O_2_ Generation

MCF-7 cells were seeded in confocal dishes for 12 h until complete adhesion (37°C, 5% CO_2_). Then, cells were treated with medium including Ce6-PLGA@PDA-PAH-DMMA NPs and Ce6@PDA-PEG NPs (Ce6 = 2 μg/ml), followed by incubating for 6 h. Next, the cells were co-cultured with DCFH-DA for 30 min and irradiated with the 660-nm laser (200 mV/cm^2^, 5 min). After that, MCF-7 cells after treatment were observed with CLSM.

## Results and Discussion

### Preparation of Ce6-PLGA@PDA-PAH-DMMA NPs

The preparation of Ce6-PLGA@PDA-PAH-DMMA NPs was illustrated in Figure 1A, which included three steps. Briefly, Ce6, a photosensitizer with poor solubility, was loaded into PLGA by nanoprecipitation (Ce6-PLGA NPs). Secondly, Ce6-PLGA NPs were coated with PDA through an oxidative polymerization reaction in an alkaline environment (Ce6-PLGA@PDA NPs), which endowed Ce6-PLGA@PDA NPs with photothermal performance and groups (amino and carboxyl groups) for further modification. Finally, Ce6-PLGA@PDA NPs were modified with PAH-DMMA charge-reversal layer through the Michael addition reaction and electrostatic interaction.

### Characterization of NPs

**FIGURE 2:**
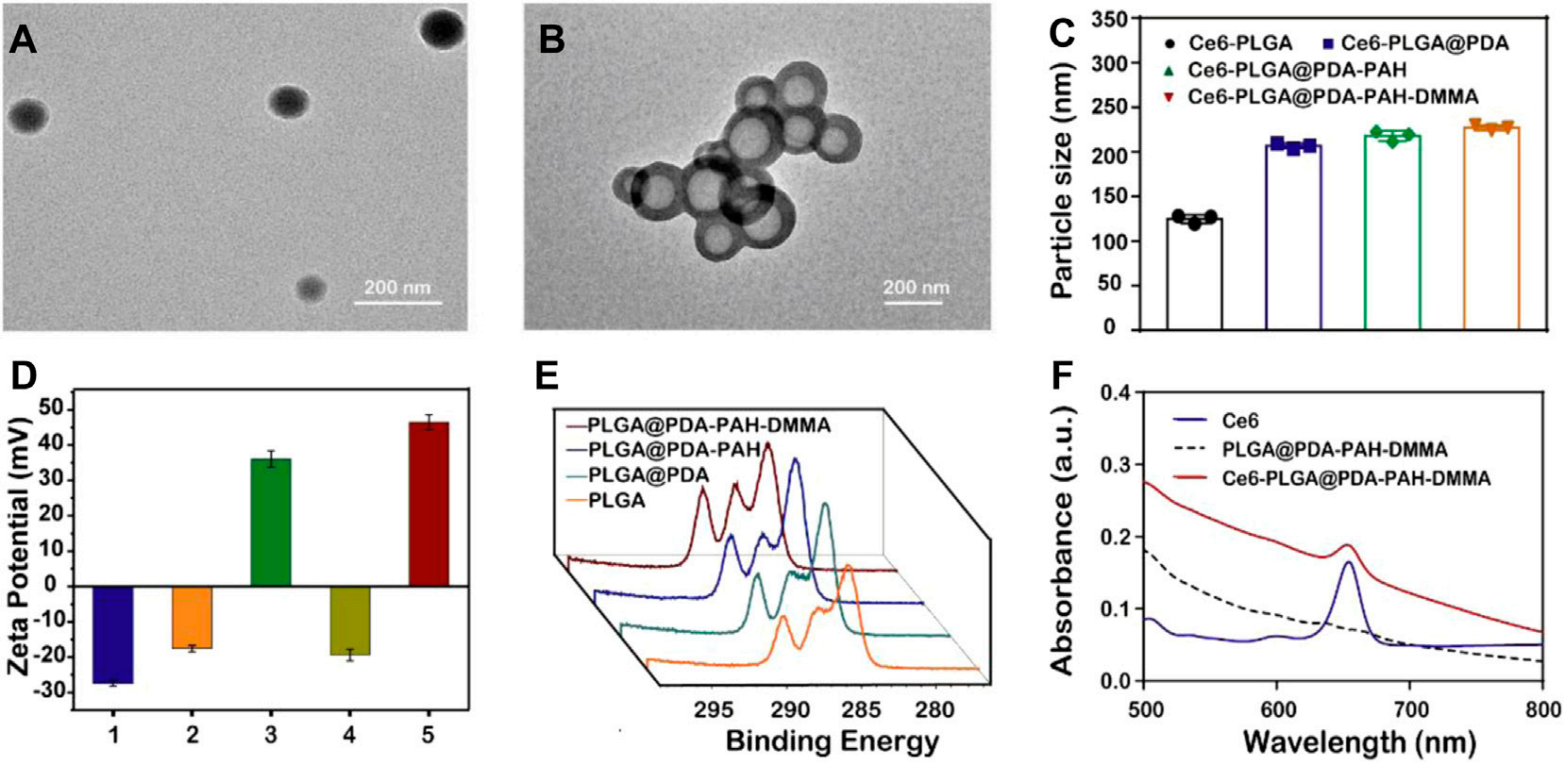
Characterization of the synthesized NPs. TEM images of **(A)** Ce6-PLGA NPs; **(B)** Ce6-PLGA@PDA-PAH-DMMA NPs. **(C)** Particle size of synthesized NPs. **(D)** Surface zeta potentials of different NPs (1, 2, 3, 4, and 5 present Ce6-PLGA NPs, Ce6-PLGA@PDA NPs, Ce6-PLGA@PDA-PAH NPs, Ce6-PLGA@PDA-PAH-DMMA NPs-pH 7.4, and Ce6-PLGA@PDA-PAH-DMMA NPs-pH 5.5). **(E)** XPS spectra of PLGA NPs, PLGA@PDA NPs, PLGA@PDA-PAH NPs, and PLGA@PDA-PAH-DMMA NPs. **(F)** UV-vis spectra of Ce6, PLGA@PDA-PAH-DMMA NPs, and Ce6-PLGA@PDA-PAH-DMMA NPs.

The morphology of Ce6-PLGA NPs and Ce6-PLGA@PDA-PAH-DMMA NPs was observed by transmission electron microscopy (TEM). Figures 2A,B show that Ce6-PLGA NPs were spherical at approximately 125 nm, but after modification, the surface of Ce6-PLGA NPs had significant coatings resulting in increased particle size of Ce6-PLGA@PDA-PAH-DMMA NPs (about 225 nm). Similarly, the increased size was also verified in the results of dynamic light scattering (Figure 2C, Supplementary Table S1). As we can see from the surface zeta potential results (Figure 2D), both Ce6-PLGA NPs and Ce6-PLGA@PDA NPs appeared as negative charges, but Ce6-PLGA@PDA-PAH NPs showed a positive potential (36.0 ± 2.3 mv) because of the cationic layer PAH. After being modified by 2,3-dimethylmaleic anhydride, the zeta potential of Ce6-PLGA@PDA-PAH-DMMA NPs became negative, increasing the circulation of the NPs. As reported, the negatively charged polymer PAH-DMMA could be hydrolyzed and become a positively charged polymer under a weakly acidic environment. To prove it, we analyzed the zeta potential of Ce6-PLGA@PDA-PAH-DMMA NPs at different pH. We found that Ce6-PLGA@PDA-PAH-DMMA NPs had a negative charge under normal physiological conditions (pH 7.4), while it had apparent charge-reversal capability from negative potential to positive potential under a mildly acidic environment. This phenomenon indicated that this nanoparticle could potentially flip into a positive charge under the weakly acidic tumor microenvironment, which may help the penetration of Ce6-PLGA@PDA-PAH-DMMA NPs in the tumor tissue.

In addition, we verified the successful loading of PDA and the charged layer on the surface of PLGA NPs by FT-IR and XPS analysis. After modifying PDA, a prominent absorption peak between 1,600 cm^−1^ and 1,530 cm^−1^ was observed, which was assigned to the overlap of the C=C resonance vibrations and the N–H bending vibrations, suggesting that the PDA layer successfully adhered to the surface of PLGA NPs (Supplementary Figure S2A). Furthermore, the results of the XPS analysis also verified it. Bands of the C–C group (∼284 eV) and C–O group (∼286 eV) intensities of PLGA@PDA NPs, PLGA@PDA-PAH NPs, and PLGA@PDA-PAH-DMMA NPs showed an increasing trend, which is attributed to the PDA layers and the successful connection of PAH and DMMA (Figure 2E). As for the N1s spectrum (Supplementary Figure S2B), the NPs appeared to significantly increase the band intensity at 400 eV. These results confirmed the successful synthesis of PLGA@PDA-PAH-DMMA NPs. The photosensitizer Ce6 had a characteristic ultraviolet absorption peak at 654 nm. Based on it, we measured the UV characteristic peak of NPs to confirm the success of Ce6-loaded. Compared to pure PLGA@PDA-PAH-DMMA NPs, Ce6-PLGA@PDA-PAH-DMMA NPs had an apparent absorption band at approximately 654 nm, indicating that Ce6 was successfully encapsulated into PLGA@PDA-PAH-DMMA NPs (Figure 2F). To test the encapsulation efficiency and loading content, we measured the free Ce6 in the supernatant after loading the drug by UV-vis absorption spectra. Results showed that the encapsulation efficiency (EE) and loading content of Ce6 were approximately 78% and 4.2%, respectively (Supplementary Figure S3).

### Photothermal Performance

**FIGURE 3:**
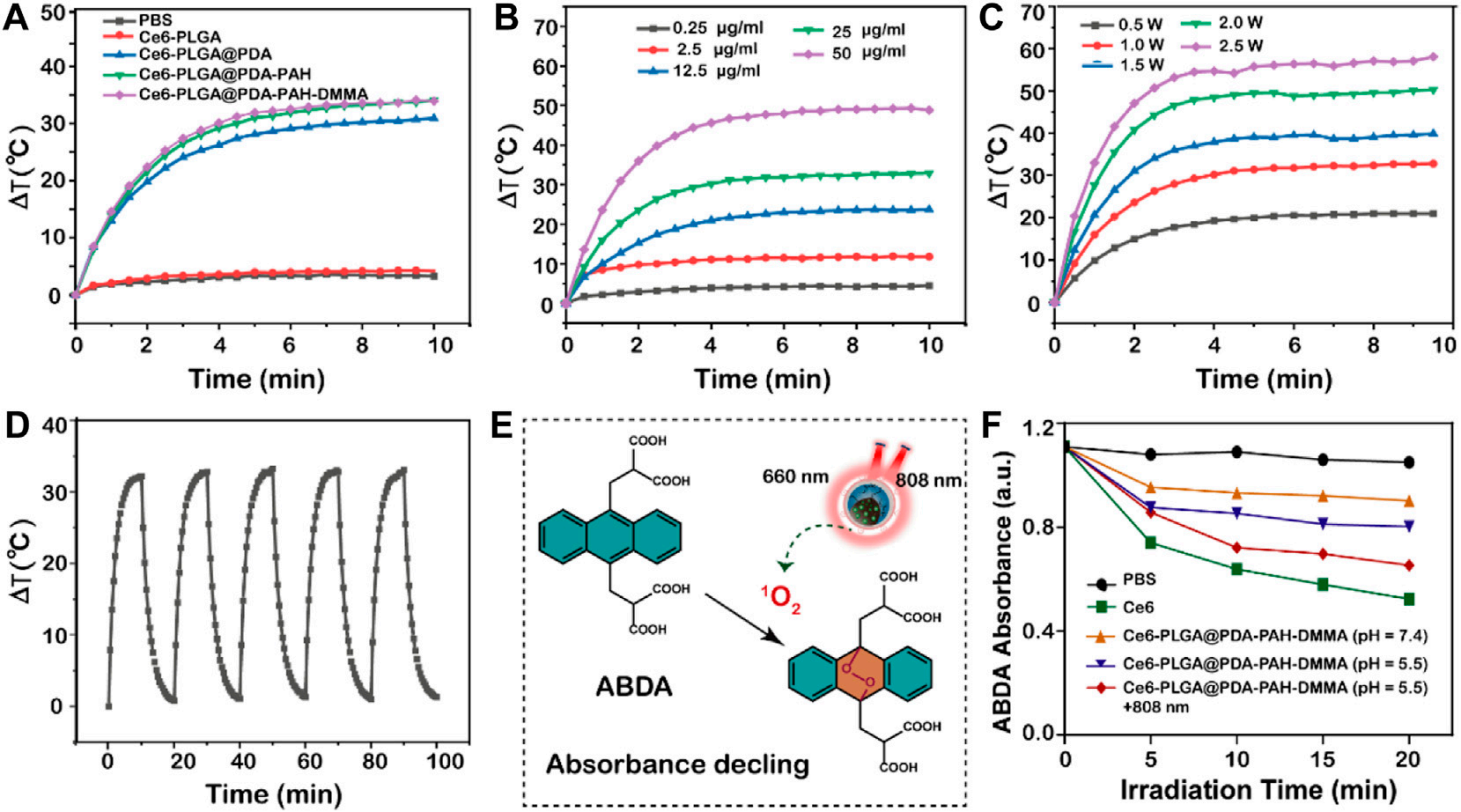
Photothermal properties. **(A)** Heating curves of different NPs after irradiating by the 808-nm laser. **(B)** Photothermal heating curves of different concentrations of Ce6-PLGA@PDA-PAH-DMMA NPs irradiated by the 808-nm laser (1.5 W/cm^2^, 10 min). **(C)** Temperature changes of Ce6-PLGA@PDA-PAH-DMMA NPs exposed to the 808-nm laser in different power densities. **(D)** Heating/cooling curves for 10 min-on–10 min-off laser irradiations (808 nm, 1.5 W/cm^2^, 5 times). **(E)** Detection mechanism of ABDA for ^1^O_2_. **(F)** Absorption spectra of ABDA for ^1^O_2_ generation in different formulations treated in different ways.

As reported, PDA had an excellent photothermal effect and could be used for photothermal therapy. To investigate the photothermal performance of NPs, we recorded the temperature of NPs under the 808-nm laser irradiation *in vitro*. We observed that the temperatures of the NPs with the PDA layer all increased rapidly by approximately 30°C in 10 min under NIR irradiation (1.5 W/cm^2^), performing excellent light and heat heating effect, while others did not (Figure 3A). Moreover, the temperature rise of Ce6-PLGA@PDA-PAH-DMMA NPs depended on nanoparticle concentration and irradiation intensity (Figures 3B,C), suggesting that we could adjust the concentration of the NPs and the irradiation intensity to meet actual application needs. Furthermore, the photothermal stability of the photothermal agent was crucial for the application of photothermal therapy. Thus, we irradiated Ce6-PLGA@PDA-PAH-DMMA NPs solution in five on/off cycles through NIR laser (2 W/cm^2^), finding no significant difference in temperature changes (Figure 3D). In summary, Ce6-PLGA@PDA-PAH-DMMA NPs had superior light-to-heat conversion performance and great light-to-heat stability.

### *In Vitro* ROS Generation

Limited by the content of Ce6 and instrument sensitivity, we indirectly reflected the release of Ce6 by measuring the ability of drug release solutions of each group to generate ROS under the 660-nm laser irradiation. To explore the ability of NPs to generate ROS (such as ^1^O_2_) *in vitro*, we used ABDA as a singlet oxygen (^1^O_2_) indicator for determining. ABDA could react with single oxygen to generate corresponding endoperoxides, causing a drop in UV absorption at 400 nm (Figure 3E). Results showed that the ABDA fluorescence intensity of Ce6-PLGA@PDA-PAH-DMMA NPs (pH 5.5 + 808 nm) declined to 65.38%, which was slightly lower than 52.3% of Ce6 and higher than other groups after irradiating with the 660-nm laser (Figure 3F). Therefore, we anticipated that Ce6-PLGA@PDA-PAH-DMMA NPs increased the release of Ce6 at pH 5.5 and irradiated with the 808-nm laser, and could effectively produce toxic.

### *In Vitro* Cellular Uptake

**FIGURE 4:**
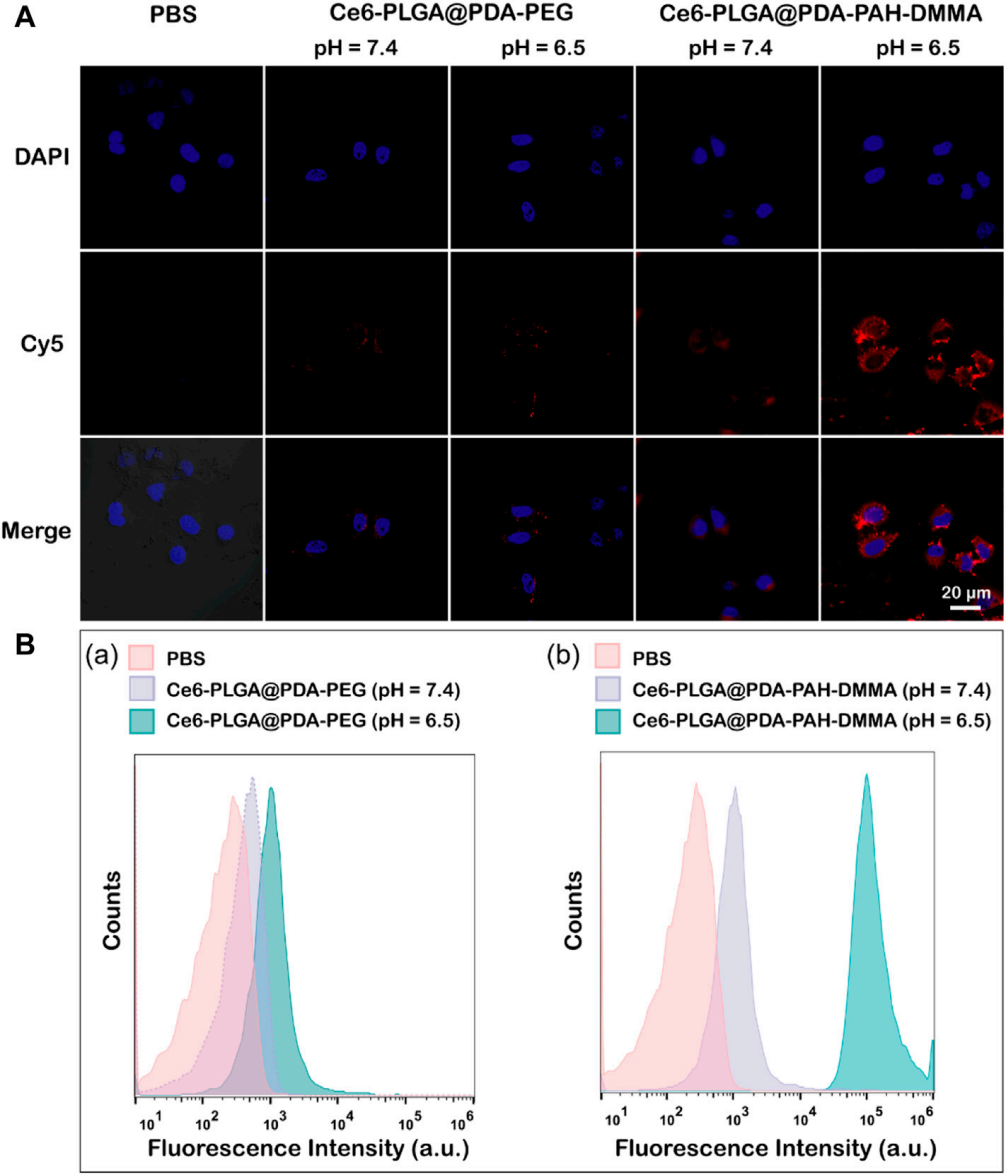
**(A)** Confocal images of MCF-7 cells cultured separately with the NPs at pH 7.4 and 6.5 for 2 h at 37°C (Blue: DAPI; Red: Cy5 fluorescence). **(B)** Flow cytometric analysis of MCF-7 cells after treating with Ce6-PLGA@PDA-PEG NPs **(A)** and Ce6-PLGA@PDA-PAH-DMMA NPs **(B)** at pH 7.4 and 6.5.

Owing to a weakly acidic environment, the surface charge of Ce6-PLGA@PDA-PAH-DMMA NPs would flip into the positive charge, which could interact with the negatively charged cell membrane of cancer cells, promoting cellular uptake. Thus, we evaluated its internalization by MCF-7 cells at pH 6.5 and pH 7.4 for 2 h. The Cy5 was used to label the Ce6-PLGA@PDA-PEG NPs and Ce6-PLGA@PDA-PAH-DMMA NPs. As revealed by confocal images, after 2 h co-incubation with MCF-7 cells, the group of Ce6-PLGA@PDA-PAH-DMMA NPs (pH = 6.5) had a strong red fluorescence signal distributed in the cytoplasm, which was much higher than it was at pH 7.4 and the groups of Ce6-PLGA@PDA-PEG NPs. This phenomenon demonstrated that Ce6-PLGA@PDA-PAH-DMMA NPs could promote the uptake of cancer cells under a weakly acidic environment (Figure 4A). At the same time, cell flow cytometry was also used to certify the internalization of Ce6-PLGA@PDA-PAH-DMMA NPs by MCF-7 cells and similar results were found. As shown in Figure 4B, we discovered that, after culturing with Ce6-PLGA@PDA-PAH-DMMA NPs for 2 h, the mean fluorescence intensity of MCF-7 cells at pH 6.5 is four times more than that at pH 7.4. In contrast, it was very weak when co-incubation with Ce6-PLGA@PDA-PEG NPs at pH 6.5 or pH 7.4.

### *In Vitro* Cellular Cytotoxicity

**FIGURE 5:**
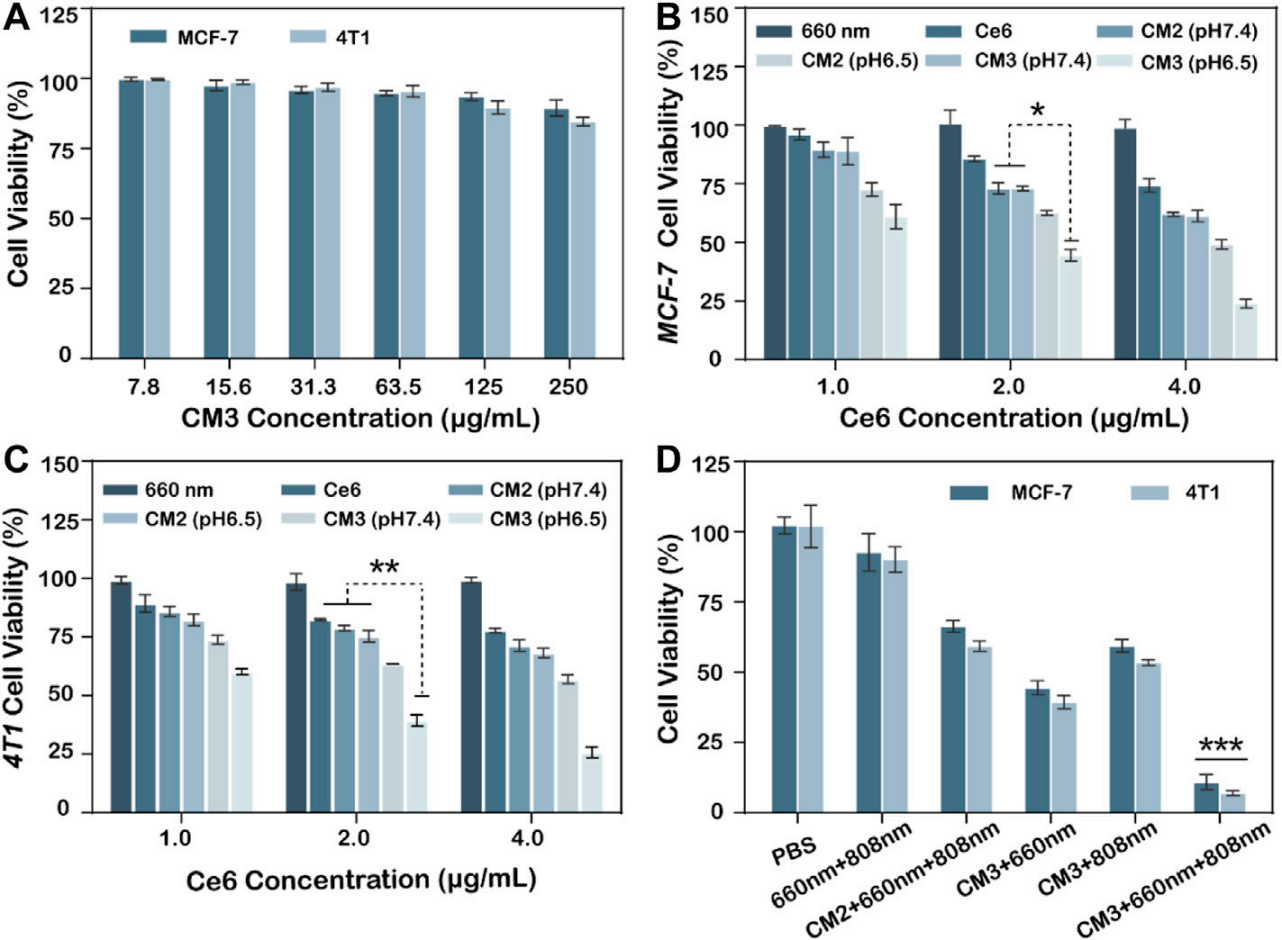
**(A)** Viability of MCF-7, 4T1 cells after treating with different concentrations of Ce6-PLGA@PDA-PAH-DMMA (CM3) without lasers. Viability of MCF-7 cells **(B)** and 4T1 cells **(C)** after incubation with Ce6-PLGA@PDA-PEG NPs (CM2) and Ce6-PLGA@PDA-PAH-DMMA NPs (CM3) at concentrations of Ce6 (1.0 μg/ml, 2.0 μg/ml, and 4.0 μg/ml) under the 660-nm laser irradiation (200 mW/cm^2^, 5 min) at pH 6.5. **(D)** Viability of cancer cells after PTT or PDT alone and PTT/PDT treatments (*t*-test, *p < 0.05, **p < 0.01, ***p < 0.001 and *n* = 3); Q value method: Q = V (a + b)/(Va + Vb—Va* Vb). V (a + b) is the inhibition rate of combined A and B drug, Va and Vb are the A and B drugs alone (Q < 0.85 is antagonism, 0.85 ≤ Q < 1.15 is additive, and Q ≥ 1.15 is synergy).

To evaluate the ability of Ce6-PLGA@PDA-PAH-DMMA NPs to inhibit MCF-7 cells and 4T1 cells, a cell counting kit (cck8) was used. As we can see from Figure 5A, the survival rate of MCF-7 and 4T1 cells were all over 85% after co-cultivation with different concentrations of Ce6-PLGA@PDA-PAH-DMMA NPs for 24 h, indicating it had excellent biocompatibility in no near-infrared radiation. On the contrary, Ce6-PLGA@PDA-PAH-DMMA NPs containing different concentrations of Ce6 inhibited the growth of the cells under the 660-nm laser irradiation (Figures 5B,C). Furthermore, after irradiation with the 660-nm laser (200 mW/cm^2^, 5 min), Ce6-PLGA@PDA-PAH-DMMA NPs had a better-killing effect on cancer cells at pH 6.5 than that at pH 7.4. Reasons for this result may be more cell uptake promoted by charge flipping and faster drug release caused by the decomposition of PDA in a weakly acidic environment. Subsequently, to explore the effect of the combined PTT/PDT therapy, we selected Ce6-PLGA@PDA-PEG NPs or Ce6-PLGA@PDA-PAH-DMMA NPs with a Ce6 content of 2 ug/ml as therapeutic agents. As shown in Figure 5D, a significantly reduced cell survival rate (MCF-7: 59.43%; 4T1: 53.46%) was observed when the cancer cells were treated with Ce6-PLGA@PDA-PAH-DMMA NPs at pH 6.5 under the 808-nm laser irradiation, which was attributed to the photothermal ability of PDA. As expected, we found that the combination therapy (Ce6-PLGA@PDA-PAH-DMMA NPs + 660 nm + 808 nm) showed the lowest viability of MCF-7 (10.9%) and 4T1 cells (7.05%), compared with other groups. The above results indicated that, under weakly acidic conditions, the ability to inhibit cancer cell growth of synergistic PTT/PDT therapy was stronger than PTT or PDT alone. Furthermore, according to the Q value method: Q = V (a + b)/(Va + Vb—Va* Vb), we obtain the following results: Q_MCF-7_ = 1.212; Q_4T1_ = 1.178. Both of them are greater than 1.15, proving that PDT and PTT are working synergistically.

### *In Vitro* ^1^O_2_ Generation

**FIGURE 6:**
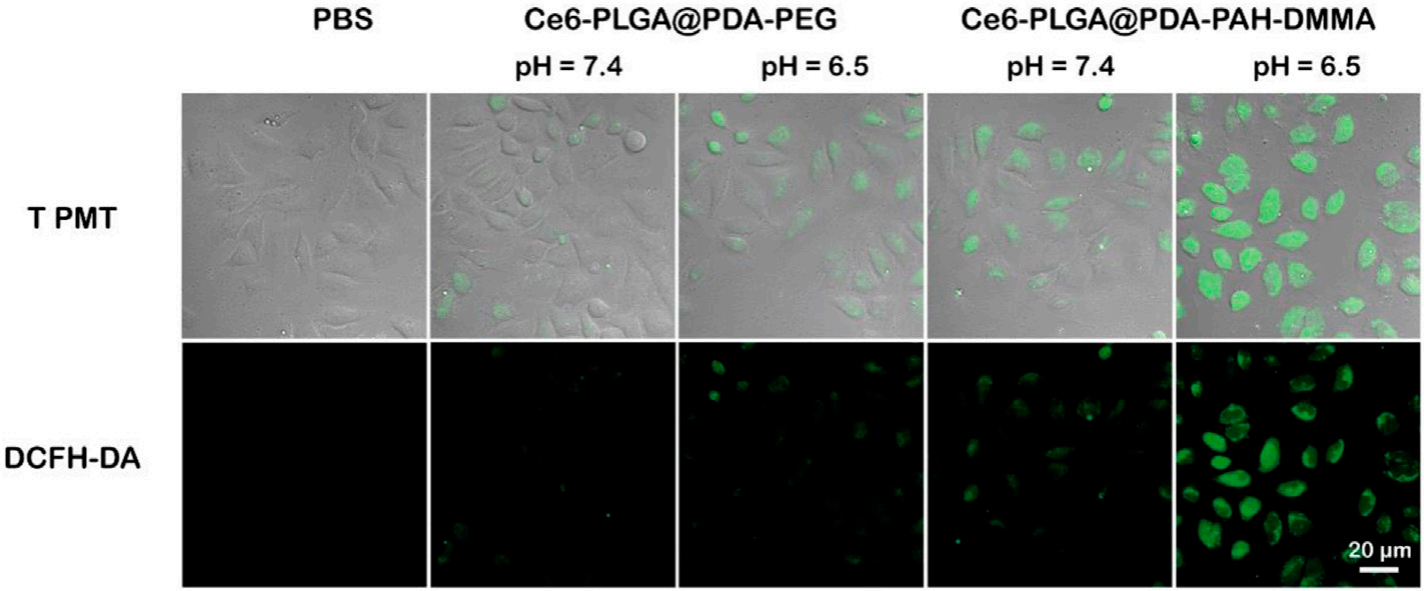
Confocal images of ROS generation by cells treated with PBS (control), Ce6-PLGA@PDA-PEG NPs, and Ce6-PLGA@PDA-PAH-DMMA NPs.

To further verify the ability of Ce6-PLGA@PDA-PAH-DMMA NPs to generate ROS during intracellular PDT, the DCFH-DA kit, a molecule that could enter cells and react with ^1^O_2_ to emit fluorescence, was used to detect the intracellular ^1^O_2_ level. The results were observed by confocal images of MCF cells incubated with different NPs at different pH values (pH 6.5 and pH 7.4) and treated with DCFH-DA for 30 min before irradiating with the 660-nm laser. As shown in Figure 6, compared with other groups, the strongest intracellular fluorescence was discovered when treated with Ce6-PLGA@PDA-PAH-DMMA NPs at pH 6.5, illustrating that most ^1^O_2_ were generated. The cells incubated with Ce6-PLGA@PDA-PEG NPs at pH 6.5 and pH 7.4 or Ce6-PLGA@PDA-PAH-DMMA NPs at pH 7.4 showed weak fluorescence. In conclusion, Ce6-PLGA@PDA-PAH-DMMA NPs could efficiently generate massive toxic ^1^O_2_ at pH 6.5, which may be attributed to the mass internalization of Ce6-PLGA@PDA-PAH-DMMA NPs by cells at pH 6.5.

## Conclusion

In conclusion, we built a pH-responsive and charge-reversal nanoplatform (Ce6-PLGA@PDA-PAH-DMMA NPs) that can enhance breast cancer therapeutic efficiency. Ce6-PLGA@PDA-PAH-DMMA NPs with a charge-reversal layer, which could maintain a negative potential at pH 7.4 and become a positive potential at the tumor extracellular microenvironment (pH 6.5), promoted their enrichment in the tumor and uptake by cancer cells. Subsequently, after irradiation with the 808-nm laser and the 660-nm laser, the photothermal effect of PDA and the ROS produced by Ce6 can synergistically damage breast cancer. *In vitro* experiments manifested that Ce6-PLGA@PDA-PAH-DMMA NPs showed increased uptake by cancer cells and overcame the limited tumor therapeutic efficacy of PTT or PDT alone. Taken together, Ce6-PLGA@PDA-PAH-DMMA NPs offer a promising approach with enhanced therapeutic efficiency for breast cancer treatment.

## Data Availability

The original contributions presented in the study are included in the article/Supplementary Material, Further inquiries can be directed to the corresponding authors.
