# Supplementary material for: pH-Sensitive and Charge-Reversal Polymeric Nanoplatform Enhanced Photothermal/Photodynamic Synergistic Therapy for Breast Cancer
Source: Front Bioeng Biotechnol. 2022 Feb 18;10:836468. doi: 10.3389/fbioe.2022.836468 (PMC8895045; doi:10.3389/fbioe.2022.836468)

***Supporting Information***

**pH-Sensitive and Charge-Reversal Polymeric Nanoplatform Enhanced Photothermal/Photodynamic Synergistic Therapy for Breast Cancer**

Wenyan Wang^a,1^, Zimu Li^a,1^, Xiaozhong Nie^b,1^, Wenfeng Zeng^a^, Yi Zhang^a^, Yimin Deng^a^, Hongzhong Chen^a^, Xiaowei Zeng^a^, Hualin Ma^c,*^, Yi Zheng^d,*^, and Nansha Gao^a,*^

*^a^ Institute of Pharmaceutics, School of Pharmaceutical Sciences (Shenzhen), Sun Yat-sen University, Shenzhen 518107, China*

*b School of Food and Drug, Shenzhen Polytechnic, Shenzhen 518055, China*

*^c^ Shenzhen Key Laboratory of Kindey Diseases, Department of Nephrology, Shenzhen People’s Hospital (The Second Clinical Medical College，Jinan University; The First Affiliated Hospital, Southern University of Science and Technology), Shenzhen 518020, China*

*^d^ Central Laboratory,University of Chinese Academy of Sciences-Shenzhen Hospital, Shenzhen 518106, China*

^1^ These authors contributed equally to this work.

^*^ Corresponding authors.

E-mail: gaonsh@mail.sysu.edu.cn (N.Gao)

E-mail: zhengyi1205@126.com (Y. Zheng)

E-mail: mahualin0796@sina.com (H. Ma)

### 1. Materials

Tri (Hydroxymethyl) Amino Methane Hydrochloride (Tris-HCl, pH8.5), D-α-Tocopherol polyethylene glycol 1000 succinate (TPGS), PLGA (LA : GA = 75:25, Mn ≈ 25000), chlorin e6 (Ce6), dopamine hydrochloride, 2,3-Dimethylmaleic anhydride (DMMA), Poly(allylamine hydrochloride) (PAH), Triethylamine, Cy5-NHS ester, dialysis bag (Mw = 3500), Dimethyl sulfoxide (DMSO), high-glucose Dulbecco's modified Eagle medium (DMEM), 2',7'-dichlorodihydrofluorescein diacetate (DCFH-DA), 4% paraformaldehyde, 4',6-diamidino-2-phenylindole (DAPI), 9,10-anthracenediyl-bis(methylene) dimalonic acid (ABDA).

### 2. Statistical Analysis

The experimental data are expressed as mean ± standard deviation (SD). Statistical analysis was performed by one-way ANOVA followed by Bonferroni test with SPSS 22.0 software. P < 0.05 was considered statistically significant.

**
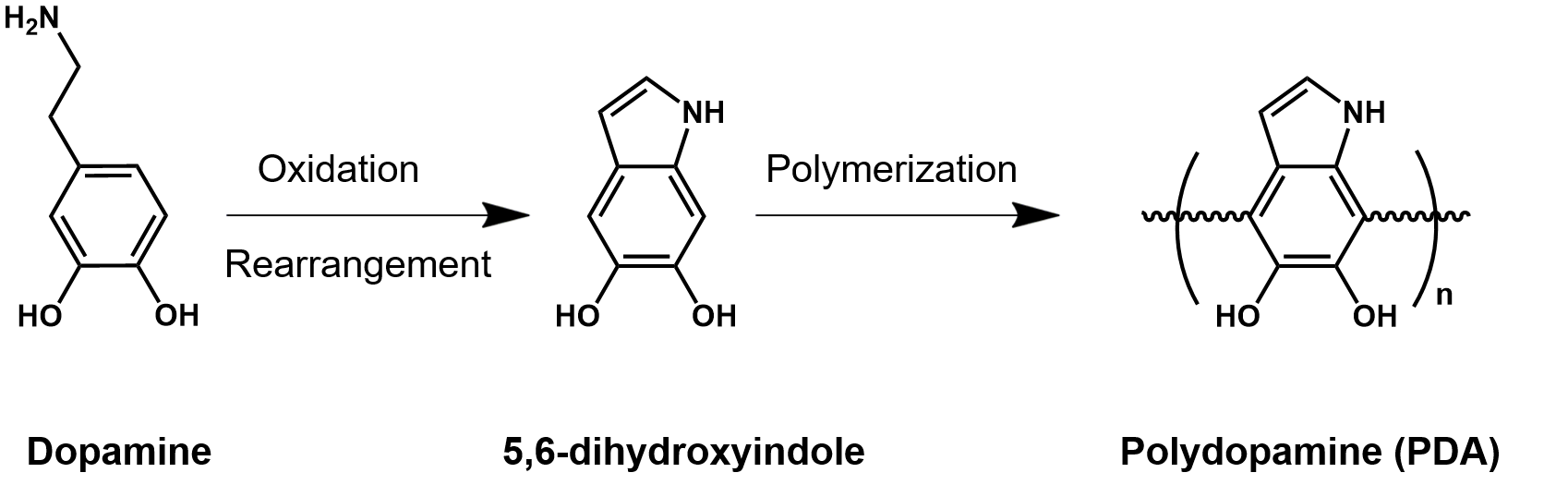
**

**Figure S1.** The mechanism of dopamine polymerization.


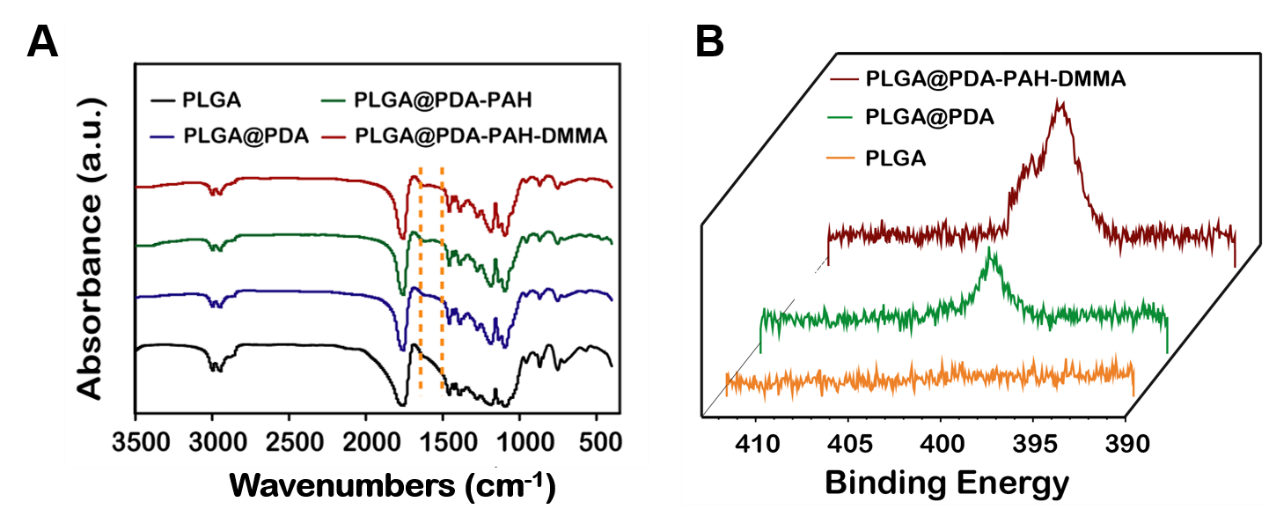


**Figure S2** (A)FT-IR of PLGA NPs, PLGA@PDA NPs, PLGA@PDA-PAH NPs, PLGA@PDA-PAH-DMMA NPs; **(B)** XPS spectra of PLGA NPs, PLGA@PDA NPs, PLGA@PDA-PAH NPs, PLGA@PDA-PAH-DMMA NPs (N1s spectrum).


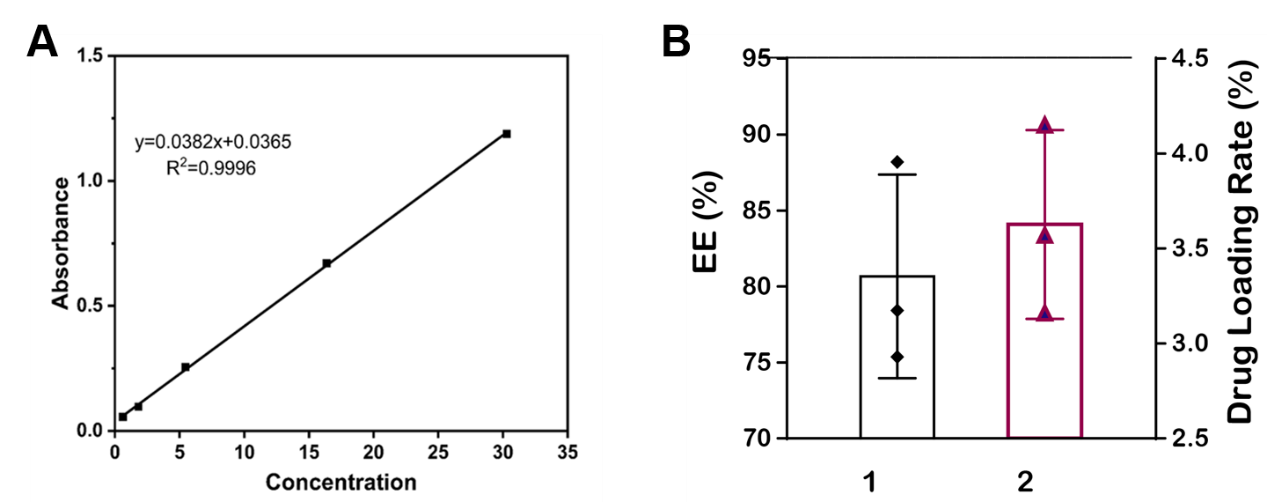


**Figure S3** **(A)** UV absorption standard curve of Ce6; **(B)** The encapsulation efficiency and loading content of Ce6-PLGA@PDA-PAH-DMMA NPs.

Table S1. DLS size distribution of Ce6-PLGA NPs, Ce6-PLGA@PDA NPs, Ce6-PLGA@PDA-PAH NPs, Ce6-PLGA@PDA-PAH-DMMA NPs.


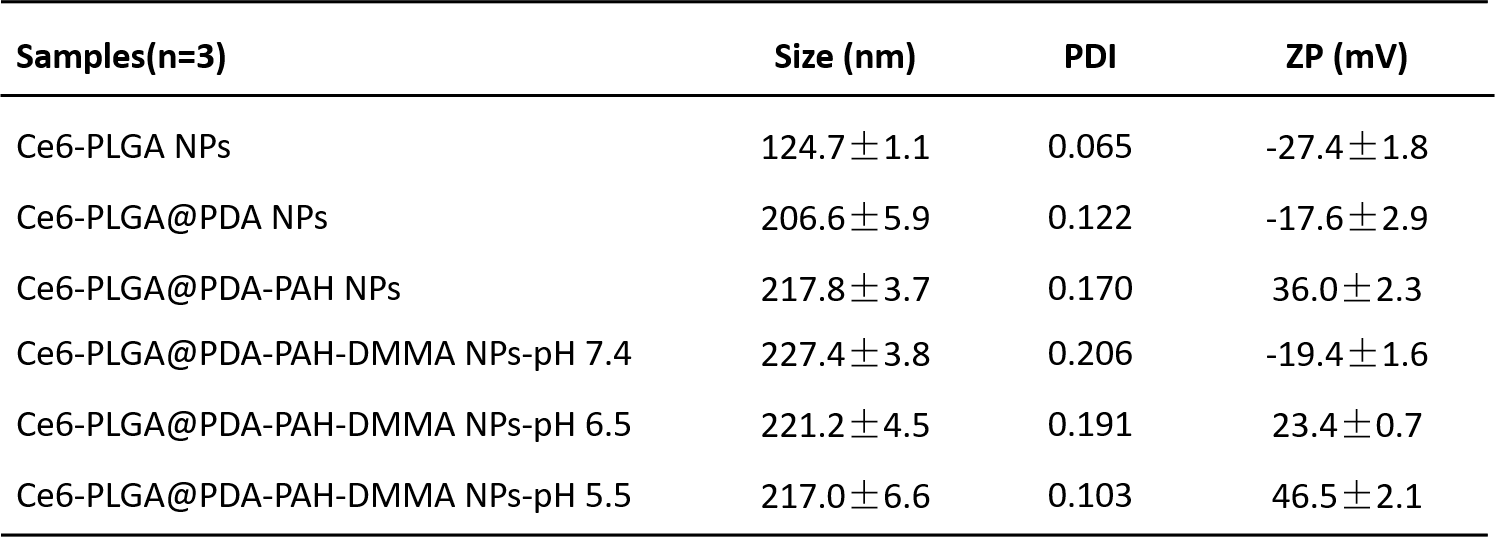

Supplement: Supplementary file 1 [file DataSheet1.docx]
